# The Brain Differentially Prepares Inner and Overt Speech Production: Electrophysiological and Vascular Evidence

**DOI:** 10.3390/brainsci10030148

**Published:** 2020-03-04

**Authors:** Franziska Stephan, Henrik Saalbach, Sonja Rossi

**Affiliations:** 1 Department of Educational Psychology, Faculty of Education, Leipzig University, 04109 Leipzig, Germany; henrik.saalbach@uni-leipzig.de; 2 Leipzig Research Center for Early Child Development, Leipzig University, 04109 Leipzig, Germany; 3 ICONE—Innsbruck Cognitive Neuroscience, Department for Hearing, Speech, and Voice Disorders, Medical University of Innsbruck, 6020 Innsbruck, Austria

**Keywords:** speech production, inner speech, overt speech, event-related brain potentials (ERPs), functional near-infrared spectroscopy (fNIRS)

## Abstract

Speech production not only relies on spoken (overt speech) but also on silent output (inner speech). Little is known about whether inner and overt speech are processed differently and which neural mechanisms are involved. By simultaneously applying electroencephalography (EEG) and functional near-infrared spectroscopy (fNIRS), we tried to disentangle executive control from motor and linguistic processes. A preparation phase was introduced additionally to the examination of overt and inner speech directly during naming (i.e., speech execution). Participants completed a picture-naming paradigm in which the pure preparation phase of a subsequent speech production and the actual speech execution phase could be differentiated. fNIRS results revealed a larger activation for overt rather than inner speech at bilateral prefrontal to parietal regions during the preparation and at bilateral temporal regions during the execution phase. EEG results showed a larger negativity for inner compared to overt speech between 200 and 500 ms during the preparation phase and between 300 and 500 ms during the execution phase. Findings of the preparation phase indicated that differences between inner and overt speech are not exclusively driven by specific linguistic and motor processes but also impacted by inhibitory mechanisms. Results of the execution phase suggest that inhibitory processes operate during phonological code retrieval and encoding.

## 1. Introduction

Speech production is not only the most sophisticated medium to impart our thoughts and to mediate cognition but also the most complex motor act that requires the integration of linguistic (i.e., lemma retrieval and selection, phonological code retrieval, and phonological encoding) and sensorimotor processes (e.g., articulatory control and feedback processes) [1,2,3,4,5,6,7]. However, speech production cannot only occur overtly in spoken output (i.e., overt speech) but also silently (i.e., inner speech). Little is known about whether inner and overt speech are processed differently and, if this is the case, which processing steps are different between overt and inner speech production. One possible approach to this question consists in examining the fine-grained temporal neural dynamics and brain areas underlying the processing of inner and overt speech during a picture-naming paradigm. To reach this goal we simultaneously applied electroencephalography (EEG) and the functional near-infrared spectroscopy (fNIRS) [8].

### 1.1. Inner Versus Overt Speech

There is an ongoing debate on whether overt and inner speech are similarly processed (i.e., both include detailed articulatory information though inner speech only lacks the production of sound) or not (i.e., inner speech does not include detailed articulatory information at all and thus contains impoverished information). The debate began with two contrary theories. Vygotsky [9] asserted that inner speech is completely different from overt speech, whereas Watson [10] proposed the difference between both is mainly quantitatively (e.g., related to loudness) but not qualitatively, that is, the same processing steps are activated in inner and overt speech. Since then, studies have tried to answer this question. Oppenheim and Dell [11] proposed a surface-impoverished hypothesis suggesting that inner speech is impoverished at the phonological level while the lexical level is intact [11,12]. This assumption was supported by slips of the tongue studies showing the absence of phonemic substitutions (e.g., reef and leef) in inner speech. In contrast, a number of studies indeed showed that inner speech contains phonological and phonetic features [13,14,15]. This suggests that inner speech is performed exactly as overt speech but without articulation. This findings can be assigned to a second class of hypotheses, the unimpoverished hypothesis. In this view, inner speech is characterized by similar phonological and lexical features but compared to overt speech it only lacks sound and movement. These hypotheses start with the view that speech execution in speech production entails multiple processing steps. In this regard, Levelt [16] proposed a speech production model, resulting from reaction time measurements in picture-naming tasks in adults, and extended over the last decades with evidence also from neuroscientific methods [6,17,18,19,20,21]. It proposes consecutive steps during overt speech production starting with lemma retrieval, and continuing with lemma selection until about 275 ms after stimulus presentation supported by middle temporal regions. Afterwards, the phonological code retrieval starts around 275 ms in middle and superior temporal regions followed by the syllabification (phonological encoding) around 355 ms in frontal regions. The subsequent phonetic encoding starts from 455 ms onwards and is supported by frontal, predominantly motor-related areas. The actual articulation starts around 600 ms.

These different steps were put under investigation by means of neuroimaging approaches such as functional magnetic resonance imaging (fMRI) [22,23,24,25,26,27], functional near-infrared spectroscopy [28], or positron emission tomography (PET) [29]. Studies show diverging results. Some studies found a greater activation of overt compared to inner speech in motor and premotor regions [22,23,24,25,29]. These findings would be consistent with the perspective, in which inner speech is performed exactly as overt speech but without articulatory activity (being in line with the unimpoverished hypothesis). However, not only motor-related areas were more active during overt speech but also other regions such as frontal regions (inferior frontal gyrus, Broca) during word generation [27] and letter naming [26] as well as middle and superior temporal regions during word reading [29], word repetition [25], word stem completion [24], and picture naming [28]. Greater temporal activations have been postulated to be associated with the perception of one’s own speech (i.e., auditory feedback) during overt production [25,28,29]. Further, a larger frontal activation of overt compared to inner speech has been postulated as a higher degree of phonological processing/encoding which is required in the aloud but not in the silent condition [27], thus supporting the surface-impoverished hypothesis. In contrast, some studies found a greater activation of inner compared to overt speech in frontal areas during word generation [26] as well as in frontal, parietal, and middle temporal regions during a verbal fluency task [30]. A larger activation of inner compared to overt speech in these regions has been postulated as the additional need for non-linguistic processing resources such as attention and inhibition. These findings imply that inner speech production cannot be simply equated to overt speech minus articulatory motor execution. It should be noted that the mentioned neuroimaging studies directly investigated the speech execution phase. Here, the semantic content of the picture to be named or word to be read or generated might impact inner and overt speech production.

### 1.2. Speech Preparation Versus Execution

The question arises whether differences between inner and overt speech still exist when no semantic content and no motor components are involved. One opportunity to study this is the use of a preparation phase, instead of examining the overt and inner speech production network directly during naming (i.e., speech execution) [3,31]. Only few studies introduced such a preparation phase. In these studies, participants only received information about how to produce the subsequent stimuli (either aloud or silently), that is, the participants did not know the content of the upcoming stimulus during this phase. Kell and colleagues [31] used an auditory instruction (mute, normal, happy), whereas Gehrig and colleagues [3] used a visual cue (square for inner speech or triangle for overt speech) that informed the participants of how to deal with the subsequently presented sentences. Both studies aimed at investigating the pre-activation of the language network by focusing on the preparation phase. During this phase, participants already knew that they were about to speak overtly or covertly afterwards when the target sentence was presented but did not know yet the content of the upcoming stimulus. Thus, the preparation and execution phase differed with respect to the absence or presence of a concrete semantic content such as a word or a picture (e.g., of a rabbit) or a whole sentence. During the execution phase, participants had to also process the semantic content, which they had to name either overtly or covertly depending on the presented cue in the preparation phase. Thus, during the preparation phase, participants had to prepare the mode of speaking (silently or aloud) but without knowing the content of the subsequently to be named stimulus. This preparation phase requires “executive control” [3,31], which refers to control and regulation of cognitive processes for goal-directed behavior that is afterwards implemented during the execution phase [3,32]. This definition of executive control is in accordance with Kell et al. [31] and Gehrig et al. [3]. Executive control is assumed as being primarily supported by frontal lobes. In particular, the prefrontal cortex plays a crucial role in regulating thoughts, perception, and behavior through the activation and inhibition of other brain regions [33,34,35]. The concept of executive control was first described as “central executive” by Baddeley and Hitch [36] and was assumed to coordinate and monitor different subsystems (the phonological loop and visuospatial sketchpad) of working memory. Another model by Norman and Shallice [37] proposed a “Supervisory Attentional System (SAS)”. This system becomes activated when automatic processes are disrupted, for example, in novel or complex situations. This attentional system controls the selection of subsequent behavior by activating or inhibiting schemas/rules. Furthermore, executive control was proposed as being related to several other subcomponents such as planning, problem-solving, reasoning, cognitive flexibility, initiation, preservation, and alteration of goal-directed behavior, as well as selecting and implementing task rules [31,38,39,40,41]. An influential taxonomy model [42] proposed three aspects of executive control: updating (i.e., information updating and monitoring), inhibitory control (i.e., inhibition of prepotent responses), and shifting (i.e., mental set shifting). Despite several models and definitions, there is relative agreement that executive control is important for human adaptive behavior that organizes thoughts in a goal-directed manner [33]. Funahashi [43] describes executive control as a result of coordinated operations required to accomplish a particular goal. As already mentioned, Kell et al. [31] and Gehrig et al. [3] specify executive control in their studies as a mechanism for controlling the selection (i.e., preparation for speaking either overtly or covertly) and implementation of this selected rule to be turned into goal-directed behavior during the execution phase. This definition is in accordance with Funahashi’s [43] description, and seems thus appropriate in the context of the preparation of inner and overt speech. We, thus, adopt this definition of executive control for our study as we used a similar design to Kell et al. [31] and Gehrig et al. [3].

Kell et al. [31] investigated the preparation and execution phase during sentence reading by means of fMRI. In particular, during the preparation phase, overt speech showed a larger activation than inner speech in bilateral prefrontal, perisylvian areas (i.e., executive, thus articulatory, system), and left planum temporale (i.e., sensory system supporting auditory feedback). In contrast, the speech execution phase elicited larger activations for overt compared to inner speech in left parieto-temporal and perisylvian regions. The authors suggested that while the auditory feedback system was already left-lateralized during the preparation phase, the articulatory system showed this lateralization only during the execution phase. Thus, the brain seems to prepare the sensory consequences of speaking well before the execution. In particular, the left planum temporale was found to be involved in auditory feedback in overt speech. Thus, the study showed that before speech is acted out and articulation is initiated, the brain controls for the sensory and motor consequences of speaking. These findings were supported by the study of Gehrig et al. [3] also using a preparation phase of sentence reading. The authors investigated oscillatory activity by means of magneto-encephalography (MEG) and found a larger left-lateralized beta-band suppression of overt compared to inner speech in articulatory motor cortex and sylvian parieto-temporal regions as well as a larger left-lateralized alpha-band suppression of overt compared to inner speech in auditory regions. This alpha-suppression was assumed to reflect the increased activity in auditory cortex relevant for auditory feedback. Furthermore, the authors suggested that the increased beta suppression in motor-related regions indicates a motor preparation process. Both studies showed that the brain prepares for the sensory and motor consequences of speaking well before a specific semantic content is given, suggesting that executive control processes are already present during the preparation phase. However, it remains speculative as to which exact subcomponent of executive control is mostly relevant to inner and overt speech. Considering the surface-impoverished hypothesis assuming that inner speech inconsistently activates phonological representations (i.e., weakened or absent), inhibitory processes might be a relevant subcomponent in this regard. Given this previous research, we expect inhibitory processes to play a key role during the preparation phase.

### 1.3. The Present Study

**Figure 1:**
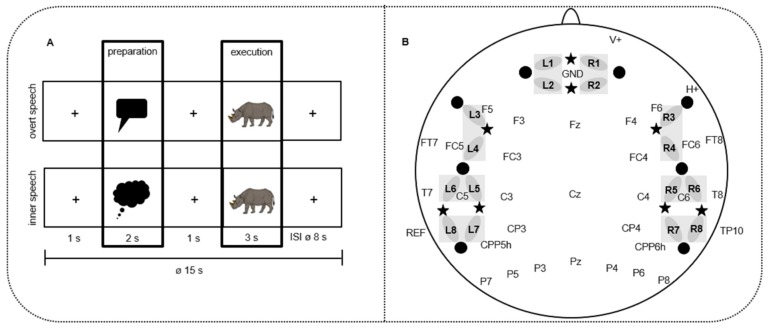
(**A**) Design of the study: event-related mini-block design. A total of 40 different colored pictures were presented twice (in inner and overt speech condition) in 16 blocks overall. Every block contained five trials of one condition. The blocks were pseudo-randomized over participants in four different versions. Each picture was cued by red speech (overt speech condition) or blue thinking (inner speech condition) bubbles. Pictures that had to be named (e.g., the rhinoceros) were taken from Rossion and Pourtois [51] with image courtesy of the authors. (**B**) Simultaneous electroencephalography (EEG) electrodes and functional near-infrared spectroscopy (fNIRS) channel placement. A total of 32 EEG electrodes (e.g., Cz); stars: 8 NIRS light emitters; dots: 8 NIRS detectors. L1-8:8 left NIRS channels; R1-8:8 right NIRS channels, resulting from the light emitter-detector arrangement. Grey bars indicate the regions of interest (ROIs) of the fNIRS channels that were used for statistical analyses.

In the present study, we aimed to investigate inner and overt speech, creating a similar preparation paradigm as in Kell et al. [31] and Gehrig et al. [3], and compared it to an actual speech execution phase but in the context of picture naming instead of sentence reading. The present study focused on a picture naming paradigm in order to have the opportunity to investigate which speech production steps differ between inner and overt speech when using only a single word instead of a sentence. In our design, we introduced a speech preparation phase presenting either a thinking or a speech bubble followed by a subsequent speech execution phase in which a concrete picture had to be named (picture-naming paradigm) either silently (inner speech) or aloud (overt speech) (Figure 1A). The design enables the investigation of executive control in the presence or absence of linguistic and motor processes. Thus, it allowed us to examine not only differences in linguistic and motor processing steps (during the execution phase), but also differences between inner and overt speech driven by executive control processes (i.e., non-linguistic features) during the preparation phase. Because neuroscientific evidence in the context of inner and overt speech preparation and production is scarce, we opted for the use of a multi-methodological approach, simultaneously assessing fNIRS as well as EEG, focusing in particular on event-related brain potentials (ERPs). ERPs are very powerful in detecting fast dynamic processes in the range of milliseconds and allow for the assessment of processes well before the initiation of articulation during speech production. fNIRS provides a better topography of neural activations but over a longer timeframe due to the sluggish hemodynamic response of the fNIRS signal [44]. Thus, these methods are complementary because they allow for the combination of good spatial and high temporal resolution [8,45]. EEG and fNIRS can be combined simultaneously at the same time in the same participant. Further advantages of this methodological combination are the soundless measurement as well as a reduced susceptibility to movement artifacts compared to fMRI [46,47]. Apart from these methodological advantages, we opted for the combination of these methods for the following reasons: (1) the activation pattern for inner and overt speech during the execution phase is not completely clear with even some contradicting results likely due to different materials, designs, and subjects put under investigation in previous studies; (2) only one fMRI study so far has investigated the preparation phase of inner and overt speech; and (3) no ERP study has focused on inner and overt speech with the intention to provide more detailed insights into the underlying processing steps in time. Therefore, the fNIRS and EEG appear to be an optimal methodological choice suitable for studying different neural signals during speech production [28,45,48,49,50].

The main question put under investigation was whether similar or different processing steps were present for inner and overt speech during the preparation and execution phase, suggesting a comparable or contrasting involvement of executive control, linguistic, and motor processes. In particular, we addressed three research questions:

(1) Do inner and overt speech differ topographically during the execution phase? On the basis of previous literature, we expected differences between inner and overt speech during the execution phase showing increased activations for overt speech predominantly in brain regions supporting phonological encoding (i.e., frontal) and auditory feedback (i.e., temporal).

(2) Which language processing steps postulated by the speech production model by Levelt [16] and Indefrey and Levelt [18] differ between inner and overt speech during the execution phase? In this regard, we further aimed at investigating the surface-impoverished and the unimpoverished hypothesis mentioned previously. Results obtained from the ERP analyses will add important timing information regarding the question of at which step (lemma retrieval, lemma selection, phonological code retrieval, phonological encoding, phonetic encoding, and articulation) do differences or similarities between inner and overt speech occur. However, due to the lack of ERP studies investigating differences between inner and overt speech during the execution phase, no concrete predictions about which steps might be different between inner and overt speech can be derived. If the surface-impoverished hypothesis [11,12] is true, differences between inner and overt speech can be expected during phonological code retrieval and encoding. If the unimpoverished hypothesis [13,14,15] is true, differences between inner and overt speech should occur at later time windows during phonetic encoding and articulation.

(3) The specific design of the present study including a preparation and execution phase aimed at investigating whether differences between inner and overt speech still exist when no semantic content (i.e., a concrete picture such as a rabbit) is presented. Is there a difference between inner and overt speech when participants only prepare the mode of speaking? In addition, are there comparable patterns of activation between the preparation and execution phase? With respect to the preparation phase, a similar difference between inner and overt speech as in the execution phase might indicate the presence of comparable inhibitory processes in both speech modes. On the basis of studies of Kell et al. [31] and Gehrig et al. [3], which found that before speech is acted out and articulation is performed, with the brain controlling for the sensory and motor consequence of speaking, we predicted differences between inner and overt speech already during the preparation phase. On the basis of the results of Kell et al. [31], we predicted a widespread bilateral activation over prefrontal and perisylvian areas larger for overt compared to inner speech, which could reflect the fact that the brain prepares the articulatory system in anticipation of the behavioral control of the planned action. Furthermore, we assumed, similar to Gehrig et al. [3] and Kell et al. [31], an increased activation for overt compared to inner speech in temporal regions during the preparation phase. If these finding results were to be the case, the involvement of auditory feedback control for the planned subsequent speech execution can be assumed. One further hypothesis that could be addressed in this regard is whether there is any need to prepare for motor consequences during the preparation phase (i.e., any difference between inner and overt speech) when the assumption is correct that inner speech does not involve articulation processes during the execution phase.

## 2. Materials and Methods

### 2.1. Participants

A total of 46 healthy native German-speaking adults (27 females; mean age: 23.2 ± SD 2.91; age range: 19–30) participated in this study. All participants gave written informed consent. Inclusion criteria were being right-handed; normal or corrected-to-normal vision; no prematurity; and no hearing, language, or neurological disorders. Handedness was assessed by the Edinburgh Handedness Inventory [52]. All 46 participants were included in the EEG analysis, whereas 11 participants had to be excluded from the fNIRS analysis due to technical artifacts.

### 2.2. Material

We developed a picture-naming task in which participants were required to name visually presented pictures. The stimulus material consisted of 40 colored drawings selected from the revised standardized set of Snodgras and Vanderwart [53] by Rossion and Pourtois [51]. We used colored pictures due to results of Rossion and Pourtois [51] finding that color information improves name agreement and naming latencies by subjects, as well as speeding up their object-recognition processes. The complete material of Rossion and Pourtois [51] includes 260 colored drawings. In order to create a homogeneous picture set, allowing an easier articulatory process, we only included two-syllabic words with a consonant–vowel onset without complex onset clusters. This restriction led to a remaining set of 60 pictures. By means of a rating, the 40 pictures with the highest naming agreement were selected. The rating was performed by 20 adults (16 females, mean age 27.7 ± SD 5.79; age range: 20–40), not participating in the neuroscientific assessment. Subjects performed a name agreement task and were instructed to name each picture as briefly and unequivocally as possible by writing the first name that came to mind. If more than one name came to mind, participants had to write each name sequentially. They were told that a name consisted of only one word. Each picture was presented on a white screen for a period of 3 s. To choose pictures with the highest agreements, we calculated the *H* value, as proposed by Snodgras and Vanderwart [53]. The statistic *H* value gives information about the distribution of names across subjects and is calculated as follows:

(1) $$H=\sum_{i=1}^{k}p_{i}\log_{2}\left(1/p_{i}\right)$$

The *H* value was calculated for each picture where *k* refers to the number of different names given to each picture and *p_i_* refers to the proportion of participants giving each name. A value of 0 indicates a perfect name agreement and an increasing *H* value shows decreasing name agreement.

Additionally, we selected pictures that align to the age of acquisition in childhood [54] as we are performing a similar study with school-aged children.

In sum, the selection criteria for the pictures included (1) a high name agreement based on the *H* value, (2) bisyllabic words in German, and (3) an age of acquisition of 60–70 months (mean 43.4 ± SD 14.7).

### 2.3. Tasks and Procedure

All participants were tested in the Lab for Cognitive Neuroscience at the Department for Hearing, Speech, and Voice Disorders of the Medical University of Innsbruck, Austria. Ethical approval was obtained from the ethical committee of the Medical University of Innsbruck. Methods were applied in accordance with the relevant guidelines and regulations and were in compliance with the Declaration of Helsinki.

The picture-naming task was programmed with Presentation Software (Neurobehavioral Systems, Inc. Berkeley, CA, USA, Version 18.1) and run on a 24″ monitor positioned at a distance of approximately 100 cm in front of the subjects. Each participant was presented colored pictures on a light grey screen. They had to name it either aloud or silently. Each trial started with a fixation cross for 1000 ms followed by a visual cue for 2000 ms for initiating the preparation phase. During this phase, either a blue thinking bubble or a red speech bubble was presented, indicating to the subjects whether they had to name the picture presented later during the execution phase aloud (overt speech) or silently (inner speech). The duration of the preparation phase was chosen on the basis of the studies of Kell et al. [31] and Gehrig et al. [3], which used a similar design as the present study. In these studies, the preparation phase lasted between 2000 and 4000 ms and the execution phase between 2000 and 3000 ms. After the preparation phase, a fixation cross for 1000 ms followed before the to-be-named picture of the execution phase was presented for 3000 ms (Figure 1A). Afterwards, a variable inter-stimulus interval (ISI) showing a fixation cross followed with a mean duration of 8000 ms (range: 6000–10000 ms). This variable ISI was introduced in order to minimize a systematic overlap of the sluggish hemodynamic response of the fNIRS signal [44].

During the overt speech in the execution phase, participants were asked to vocalize the words as softly as possible in order to reduce movement-related artifacts but loud enough that the experimenter could hear their responses. During inner speech, participants were instructed to speak the word silently in one’s mind without moving their lips. Accuracy of overt speech was 99.62%, indicating that they were able to perform the task very well.

The task paradigm was organized in a mini-block design. Each mini block consisted of five pictures in succession, corresponding to the same condition (inner/overt speech). In total, 16 blocks were presented, resulting in the presentation of 80 trials (40 inner and 40 overt speech). This means that each picture was presented twice: once in the overt speech condition and once in the inner speech condition. Blocks were organized in four different pseudo-randomization versions including maximally four blocks of the same condition in succession. This arrangement resulted in an event-related mini-block design.

Before EEG and fNIRS measurements, a practice session with 10 randomized items (five in overt and five in inner speech) was performed to familiarize the participants with the task. Participants were asked to avoid body movements during the measurements. All overt responses were logged by the experimenter. The experiment lasted 20 min in total.

### 2.4. NIRS/EEG Data Recordings

#### 2.4.1. fNIRS Data Recording

Although EEG bears the potential to detect fast changes in the range of milliseconds, fNIRS measures the changes in the concentration of oxy-hemoglobin (oxy-Hb) and deoxy-hemoglobin (deoxy-Hb) for gaining a better localization. fNIRS is suitable for monitoring overt speech because of its reduced sensitivity towards movement artifacts and because it has no acoustic noise such as fMRI, which could affect a language production study [28]. Physiologically, fNIRS measures an enhanced neural activation in a brain area elicited by an increase in regional cerebral blood flow and an increase in oxygen demand [44].

We used a NIRScout system (NIRx Medizintechnik GmbH, Berlin, Germany) measuring light attenuation at 760 and 850 nm in a cw-mode with a sampling rate of 7.81 Hz. The locations for eight light emitters and eight light detectors were arranged, covering prefrontal (PREF: L1-L2; R1-R2), frontal (FRONT: L3-L4; R3-R4), temporal (TEMP: L5-L6; R5-R6), and temporo-parietal (TPAR: L7-L8; R7-R8) brain regions (Figure 1B). An inter-optode-distance of 3.5 cm was chosen [45]. A modified EEG cap allowed for simultaneous EEG and fNIRS recordings (see Figure 1B).

#### 2.4.2. EEG Data Recording

EEG was recorded with 32 active electrodes placed in an elastic cap (actiCAP, Brain Products, Gilching, Germany) by using the BrainAmp EEG amplifier and Brain Vision Recorder software (Brain Products, Gilching, Germany). The electrodes were placed according to the 10-20 placement system of the “American Electroencephalographic Society Guidelines for standard electrode position nomenclature“ [55] at the following positions: F5 / F3 / FT7 / FC5 / FC3 / T7 / C5 / C3 / CPP5H / CP3 / P7 / P5 / P3 / F6 / F4 / FT8 / FC6 / FC4 / T8 / C6 / C4 / CPP6H / CP4 / P8 / P6 / P4 / Fz / Cz / Pz / F10 / Fp2 / TP10 / TP9 and AFz (Figure 1B). The vertical electro-oculogram (VEOG) was recorded from the Fp2 (V+), and the horizontal electro-oculogram (HEOG) was recorded from F10 (H+). Electrode positions were equally distributed over the scalp. The EEG was online referenced to the left mastoid at position TP9 and offline re-referenced to averaged mastoids including the left and right mastoids (TP10). Electrode impedance was kept below 5 kΩ. The EEG signal was digitized with 0.016 Hz to 450 Hz. The ground electrode was positioned at AFz.

### 2.5. Data Analyses

#### 2.5.1. fNIRS Data Analyses

fNIRS data analysis was performed using a MATLAB (MathWorks, Inc., Natick, MA, USA, Version R2018a)-based program nilab2 (written by Stefan Paul Koch, Charité University Medicine, Berlin, Germany). We analyzed fNIRS data on the basis of the modified Beer-Lambert Law [56] per subject and per phase (preparation, execution). In a manual artifact correction, artifacts were selected and corrected by a linear interpolation approach. To attenuate high frequency artifacts mainly resulting from the heart beat, NIRS data were low-pass filtered using a third order Butterworth filter at 0.4 Hz. A general linear model (GLM) including inner and overt speech as separate boxcar-predictors was applied using a canonical hemodynamic response function (HRF) peaking at 5 s [57]. The model provided beta values for each condition and each hemoglobin, which were fed into statistical analyses. Finally, grand averages were calculated across participants.

Statistical analyses were performed over four left- and four right-hemispheric ROIs. The ROIs were created by averaging two channels per region, allowing for anterior–posterior differences of the responses: PREF: prefrontal (L1/L2; R1/R2; L = left; R = right), FRONT: frontal (L3/L4; R3/R4), TEMP: temporal (L5/L6; R5/R6), and TPAR: temporo-parietal (L7/L8; R7/R8) (Figure 1B). We performed a four-factorial repeated measure ANOVA (CONDITION*PHASE*REGION*HEMISPHERE). The repeated measure ANOVA was separately performed for oxy-Hb and deoxy-Hb. In analogy to the EEG, the ANOVA included the within-subject factor CONDITION (overt versus inner speech), PHASE (preparation versus execution), REGION (PREF versus FRONT versus TEMP versus TPAR), and HEMISPHERE (left versus right). Significance level was assumed at *p*<0.05. Whenever the interaction between CONDITION with PHASE and/or REGION and/or HEMISPHERE reached significance, post-hoc *t*-tests were performed by applying a Bonferroni correction. We applied corrected significance according to Greenhouse and Geisser [58] whenever the degrees of freedom exceeded 1.

Typically, hemodynamic responses to cortical neural activations are evidenced by an increase in oxy-Hb and a decrease in deoxy-Hb [44].

#### 2.5.2. EEG Data Analyses

EEG data were analyzed by using the Brain Vision Analyzer 2 (Brain Products, Gilching, Germany) software. Recordings were offline filtered with a 30 Hz low-pass Butterworth zero-phase filter (slope: 12 dB/oct). Data were segmented into 1200 ms epochs (−200 ms to 1000 ms), where 0 ms represents the picture onset. Before averaging, ocular correction [59] and manual artifact rejection was conducted. After artifact rejection, 89.2% (range: 41.3%–99.7%) of overt speech stimuli during preparation phase, 89.7% (range: 58.1%–100%) of inner speech stimuli during preparation phase, 88.4% (range: 39.3%–100%) of overt speech stimuli during execution phase, and 91.5% (range: 64%–100%) of inner speech stimuli during execution phase entered final statistical analyses. In the next step, data were re-referenced to averaged mastoids and a pre-stimulus-onset baseline of 200 ms was applied. Afterwards, trials were averaged per condition, participants, and electrodes, and finally a grand average across participants was performed.

Four time windows entered the ANOVAs: 100–200 ms, 200–300 ms, 300–500 ms, and 500–600 ms. These were chosen on the basis of a 50 ms analysis, in which running paired-sample *t*-tests between overt and inner speech were performed from 100 to 600 ms in 50 ms consecutive segments. The time range for this 50 ms analysis was selected due to visual inspection of grand averages and based on previous evidence of temporal correlates in overt speech production [6,17,60,61]. The end point of this analysis at 600 ms was selected because afterwards motor execution and thus contamination with movement artifacts in the EEG starts [6].

Statistical analyses comprise 12 regions of interest (ROI) over the left and right hemisphere including two electrodes each: F3-FC3, F5-FC5, C3-CP3, C5-T7, CPP5H-P3, P5-P7; right frontal: F4-FC4, F6-FC6, C4-CP4, C6-T8, CPP6H-P4, P6-P8. Additionally, the three midline electrodes (Fz, Cz, and Pz) were analyzed separately.

Four-factorial repeated measure ANOVAs were separately performed for the three selected time windows. These ANOVAs included the within-subject factors CONDITION (overt versus inner speech), PHASE (preparation versus execution), REGION (six lateral ROIs/three midline electrodes), and HEMISPHERE (left versus right). Significance level was assumed at *p* < 0.05. Whenever the interaction between CONDITION with PHASE and/or REGION and/or HEMISPHERE reached significance, post-hoc *t*-tests were performed by applying a Bonferroni correction. We applied corrected significance according to Greenhouse and Geisser [58] whenever the degrees of freedom exceeded 1.

## 3. Results

### 3.1. fNIRS Results

**Figure 2:**
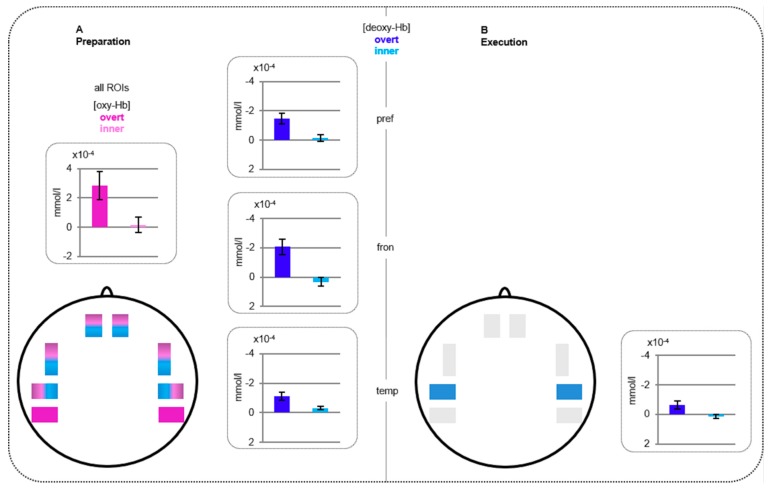
Functional near-infrared spectroscopy (fNIRS) results. Statistically significant differences between overt versus inner speech. (**A**) Beta-values for the preparation phase merged over all regions for oxy-hemoglobin (oxy-Hb; purple) and prefrontal, frontal, and temporal regions for deoxy-hemoglobin (deoxy-Hb; blue). (**B**) Beta-values for the execution phase at temporal regions for deoxy-Hb. Please note that a more positive value for oxy-Hb and a more negative value for deoxy-Hb (both plotted upwards here) are indications of increased activations.

Figure 2 provides beta values for all statistically significant interactions for the execution and preparation phase.

Oxy-Hb: The repeated measure ANOVA revealed a significant interaction CONDITION*PHASE (*F*_(1,34)_ = 4.81, *p* = 0.035) indicating a larger activation for overt compared to inner speech for the preparation phase (*t*_(34)_ = 2.74, *p* = 0.010).

Deoxy-Hb: The ANOVA showed a significant main effect of CONDITION (*F*_(1,34)_ = 19.21, *p* < 0.0001) as well as a significant interaction CONDITION*PHASE (*F*_(1,34)_ = 12.84, *p* = 0.001) and CONDITION*PHASE*REGION (*F*_(3,102)_ = 5.69, *p* = 0.005). Post-hoc testing of the three way interaction indicated a larger activation for overt compared to inner speech at prefrontal (*t*_(34)_ = −3.96, *p* < 0.0001), frontal (*t*_(34)_ = −3.42, *p* = 0.002), and temporal (*t*_(34)_ = −3.55, *p* = 0.001) regions for the preparation phase as well as at temporal regions (*t*_(34)_ = −3.03, *p* = 0.005) for the execution phase.

### 3.2. EEG Results

**Figure 3:**
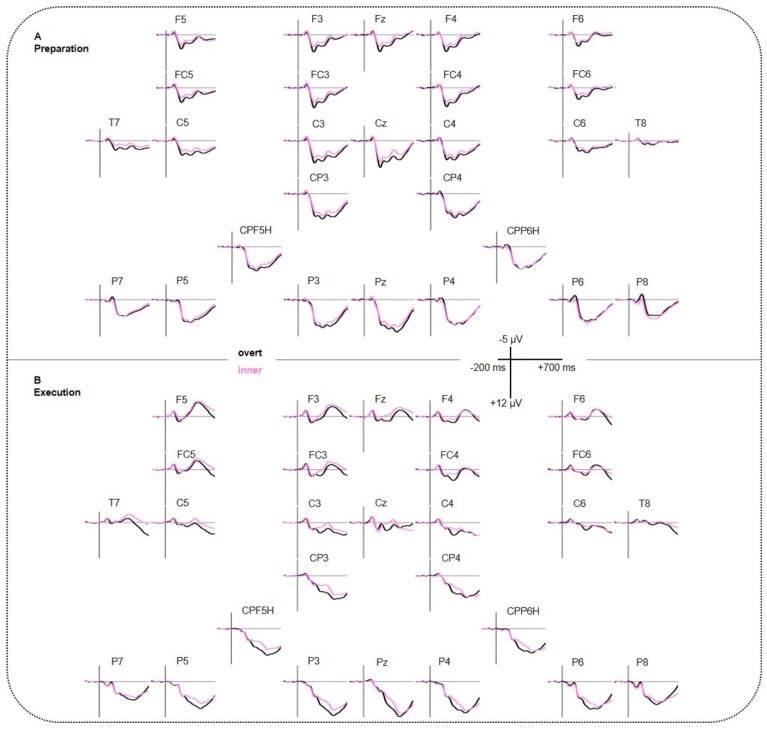
Event-related brain potentials (ERP) results. (**A**) Grand averages for the preparation phase. (**B**) Grand averages for the execution phase. Negative polarity is plotted upward.

Figure 3 illustrates ERP grand averages. It shows the comparison between the conditions overt versus inner speech for all electrodes. An 8 Hz low-pass filter (Butterworth Zero Phase Filter, high cutoff: 8 Hz, 12 dB/oct; integrated in Brain Analyzer 2, Brain Products, Gilching, Germany) was applied for presentation purposes only. Table 1 reports the significant main effects and interactions of the ANOVAs for the three time windows. For answering our research question, we only reported main effects and interactions with condition (overt vs. inner speech) as factor. Numerical results of post-hoc testing is reported in the appendices for the time windows 200–300 ms and 300–500 ms (Tables S1 and S2).

100–200 ms: The ANOVA yielded a significant interaction CONDITION*REGION for lateral electrodes resulting in a larger negativity for overt compared to inner speech at bilateral parietal regions P5P7P6P8 (*t*_(45)_ = 4.146, *p* < 0.001). Post-hoc *t*-tests for the interaction CONDITION*REGION for midline electrodes were non-significant after applying a Bonferroni correction.

200–300 ms: The ANOVA showed a significant main effect of CONDITION (lateral and midline) as well as significant interactions CONDITION*REGION (lateral) and CONDITION*PHASE*REGION/ELECS (lateral and midline). Post-hoc *t*-tests for the three-way interaction revealed that, for the preparation phase, a larger negativity for inner compared to overt speech at midline frontal and central electrodes (Fz, Cz) as well as at bilateral fronto-central and centro-temporal regions (F3FC3F4FC4, F5FC5F6FC6, C5T7C6T8). See Supplementary Table S1 for detailed information.

300–500 ms: The ANOVA showed a significant main effect of CONDITION (lateral and midline) as well as significant interactions CONDITION*REGION (lateral), CONDITION*PHASE*REGION (lateral), and CONDITION*REGION*HEMI (lateral). Post-hoc *t*-tests for CONDITION*PHASE*REGION revealed a larger negativity for inner compared to overt speech for the execution phase at centro-parietal and parietal regions (C3CP3C4CP4, CPP5HP3CPP6HP4, P5P7P6P8). Post-hoc testing for CONDITION*REGION*HEMISPHERE showed a larger negativity for inner compared to overt speech at right fronto-central, left centro-temporal, and bilateral centro-parietal regions (F4FC4, C5T7, C3CP3, C4CP4, CPP5HP3, CPP6HP4, P5P7). See Supplementary Table S2 for detailed information.

500–600 ms: The ANOVA revealed a significant interaction CONDITION*REGION*HEMI (lateral) and CONDITION*PHASE*ELECS (midline). Post-hoc *t*-tests for both interactions were non-significant after applying Bonferroni correction.

## 4. Discussion

The present study aimed to investigate inner and overt speech during the preparation of a subsequent speech production and during the actual execution of speech. To reach this goal, we presented participants with colored pictures that had to be named aloud (overt speech) or silently (inner speech). A neutral cue (a speech or thinking bubble) during the preparation phase indicated whether an overt or inner speech output was required during the following execution phase. We applied electroencephalography (EEG) and functional near-infrared spectroscopy (fNIRS) simultaneously in order to identify fast dynamic mechanisms by means of event-related brain potentials (ERPs) and involved brain areas. In the following section, we report and interpret the findings with respect to the three research questions put under investigation.

### 4.1. Brain Areas Underlying Inner and Overt Speech in the Speech Execution Phase

During the execution phase, overt speech was more active than inner speech in bilateral temporal regions. Some studies assume temporal regions to be involved in lemma retrieval as well as phonological code retrieval [6]. Other studies, however, found temporal regions to be involved in monitoring of motor output and thus resembling auditory feedback control processes [6,25,28,29]. Because hemodynamic responses in fNIRS are sluggish [44] compared to EEG and thus are expected to measure later processing stages of speech production such as the actual articulation, the bilateral temporal effect we found might be more attributable to the auditory feedback interpretation rather than to the direct processing of phonological code retrieval assumed to occur earlier in time around 300 ms [6]. These findings are in line with results obtained by means of fMRI in Kell et al. [31].

### 4.2. Timing Characteristics of Inner and Overt Speech in the Speech Execution Phase

In order to better identify the speech production process stage when inner and overt speech differ, the EEG was simultaneously assessed by fNIRS. ERP results between 100 and 200 ms showed a larger negativity for overt compared to inner speech at parietal areas, irrespective of phase. Such posterior negativities around 100 ms (N100) have previously been shown to be associated with perceptual processing [6,62]. This visual evoked potential in previous studies usually elicited a larger negativity at about 150 ms at posterior regions and was interpreted as color selection [63,64]. Furthermore, studies found a similar negativity to be larger for attended visual stimuli [64,65]. In our experiment, during the preparation phase, the different colors of the speech (blue) and thinking bubble (red) might have initiated a color selection process, thus supporting the former assumption. Why a blue color should have attracted more attention and thus supporting the latter assumption, however, remains speculative. However, because a similar early effect was observed not only during the preparation phase but also during the execution phase, a pure color selection process cannot be the sole driving force, as pictures to be named in the execution phase were of different colors. Thus, more probably, a more basic visual perceptual mechanism intertwined with attentional processes seemed to be at work here. However, why overt speech induced a larger negativity than inner speech at this early time window still remains unclear and would be interesting to address in future studies.

At later time windows of the execution phase, ERP results showed a larger negativity for inner rather than overt speech over fronto-central, centro-temporal, centro-parietal, and parietal regions starting at 300 ms. From a temporal perspective of linguistic processing, Indefrey [6] proposed a speech production model describing several steps in time. The model proposed lemma retrieval and selection within the first 275 ms, followed by phonological code retrieval (275–355 ms) and phonological encoding (355–455 ms). Our results did not show differences between inner and overt speech before 300 ms, which indicates similar processing mechanisms during these early steps. A larger negativity for inner speech compared to overt speech occurred from 300 ms onward, and thus indicated differences between inner and overt speech during phonological code retrieval and encoding [6,26,29,30,66]. These results fit very well with conclusions of behavioral studies of slips of the tongue, showing that inner speech does not just lack in articulation, but that it is also impoverished at the phonological level (surface-impoverished hypothesis) [11,67]. Although Levelt [68] proposed a serial activation of speech production in which lemma retrieval and selection precede phonological processes, there are also other prominent models assuming more parallel mechanisms at work. Dell and O’Seaghdha [69] suggested a bidirectional interaction between lexical and phonological processes. For example, if “cat” is the target word to be read, “dog” (i.e., semantically related), “rat” (i.e., semantically and phonologically related), and “mat” (i.e., phonologically related) are also activated at the stage of lexical and phonological processing. There is an interaction because “cat”, “dog”, and “rat” share semantic features (e.g., four legs) and “cat”, “rat”, and “mat” share phonological features that also get activated. Both models could account for our findings in the 300-500 ms time window, thus both phonological and lexical processes might interact at this stage. Considering the surface-impoverished hypothesis, assuming that inner speech inconsistently activates phonological representations, inhibitory processes might be a relevant mechanism in this regard. Rodriguez-Fornells et al. [66] performed a go/no-go task in which pictures were presented and had to be classified according to two different semantic and phonological categories each, finding an increased negativity for no-go-trials compared to go-trials in a similar time window. They interpreted this increased negativity as reflecting inhibitory processes. Interestingly, such inhibitory processes seem to occur also in a more linguistically oriented context before articulation. Thus, it seems plausible to assume that also our increased negativity for inner speech reflects inhibition. In order to investigate whether such inhibitory processes are also present without a linguistic context, we introduced a preparation phase prior to naming.

### 4.3. Inner and Overt Speech in the Preparation Versus Execution Phase

fNIRS results revealed a larger activation for overt compared to inner speech, widespread over bilateral prefrontal to parietal regions during the preparation phase. In line with our results, some studies primarily investigating the speech execution phase during differential tasks also found a larger activation for overt compared to inner speech over frontal, temporal, and parietal areas [25,26,27,28,29]. A larger activation of overt speech, especially in prefrontal and frontal regions, was assumed to reflect a greater effort to plan and control motor processing necessary for producing overt speech, as well as increased phonological lexical processing, particularly in the aloud condition where a concrete output has to be produced [26,27]. Notably, these activations already take place during the preparation phase, which is not contaminated with semantic content or motor execution per se. Sakai and Passingham [70] found the prefrontal cortex to also be involved in preparatory processes for a subsequent phonological and semantic task execution. Furthermore, Kell et al. [31] also found a widespread bilateral activation over prefrontal and perisylvian areas in their preparation phase, suggesting that the brain prepares the executive system in advance. Thus, our results show that the brain prepares the subsequent speech execution differentially for inner and overt speech. Similar to Gehrig et al. [3] and Kell et al. [31], our study also revealed an increased activation for overt compared to inner speech in temporal regions already during the preparation phase. This might suggest the involvement of auditory feedback control for the planned subsequent speech execution. This means, the brain prepares for the sensory and motor consequences of speaking [3,31].

EEG results of the preparation phase showed a sustained larger negativity for inner compared to overt speech over frontal, centro-temporal, centro-parietal, and parietal regions from 200 ms up to 500 ms. In go/no-go paradigms, a larger negativity around 200 ms (N200) with a fronto-central distribution was previously found in no-go trials, whereas go-stimuli showed a larger positivity (P200). The N200 was related to inhibitory processes [71,72]. In our study, a similar N200 component was found, being larger for inner compared to overt speech, and thus possibly reflecting increased inhibitory processes for the subsequent execution phase already during the preparatory phase. Strikingly, these neural processes already differentiated between inner and overt speech when no semantic content was given and no actual response was required. The N200 and P200 extended beyond 200 ms and showed a similar direction of effects until 500 ms. A late negativity was also found to reflect inhibitory control processes in previous studies [73,74]. As mentioned earlier, the studies of Kell et al. [31] and Gehrig et al. [3] showed that the preparation phase included executive control because the brain prepares for the sensory and motor consequences of speaking well before a specific linguistic content is given. Our experiment was based on the design of Kell et al. [31] and Gehrig et al. [3]. Thus, also in our study, executive control seemed to play a key role during the preparation phase in order to control for the subsequent overt and inner speech output. Because inner speech does not involve articulation processes during the execution phase, there is no need to prepare for such motor consequences during the preparation phase. As a consequence, the larger negativity for inner compared to overt speech thus reflects inhibitory mechanisms. Moreover, these results indicate that the speech production network pre-activates and respectively pre-inhibits relevant processes in anticipation of linguistic processing for overt and inner speech in order to generate the appropriate output [3,31], and thus employs comparable inhibitory mechanisms in both phases.

## 5. Conclusions

The present study demonstrated that the brain successfully differentiates between inner and overt speech. The brain prepares these processes relevant for subsequent speech execution already at an early stage when no semantic context is present. Thus, the differences between inner and overt speech seem to not be exclusively driven by specific linguistic and motor processes but are also impacted by different degrees of executive control (i.e., inhibition). Furthermore, we could specify that not only motor processes are inhibited in inner speech but that phonological code retrieval and encoding are also affected. This finding supports the surface-impoverished hypothesis. Moreover, findings clearly indicated a benefit of a multi-methodological approach assessing specific processing steps by means of different temporal and topographical resolutions. Although we replicated some neuroimaging results in a modified picture naming paradigm by means of fNIRS, the simultaneous application of fNIRS and EEG provided clearer insights concerning the exact timing of involved mechanisms, in particular during the execution phase. Having considered only the fNIRS results, we would have concluded that auditory feedback processes prevail during speech execution. However, the additional EEG results provided evidence that inhibitory processes also take place before the actual articulation. Finally, these results raise important questions such as whether inner speech is processed differentially from overt speech also in children’s brains, and whether inner speech is impaired in individuals who have disorders of overt speech, such as patients suffering from stuttering.

## Figures and Tables

**Table 1:** Electroencephalography (EEG) results of the repeated measure ANOVAs for all time windows.

Effect	100–200 ms	200–300 ms	300–500 ms	500–600 ms
Lateral ROIs				
cond	ns	(1,44):4.35/0.043	(1,44):14.35/<0.001	ns
cond*phase	ns	ns	ns	ns
cond*region	(5,22):14.98/<0.001	(5,22):9.83/<0.0001	(5,22): 6.11/0.003	ns
cond*phase*region	ns	(5,22):10.05/<0.001	(5,22):6.86/<0.001	ns
cond*hemi	ns	ns	ns	ns
cond*phase*hemi	ns	ns	ns	ns
cond*region*hemi	ns	ns	(5,22): 3.69/0.012	(5,22):7.91/<0.001
cond*phase*region*hemi	ns	ns	ns	ns
Midline ROIs				
cond	ns	(1,44):8.15/0.007	(1,44):28.68/<0.0001	ns
cond*phase	ns	ns	ns	ns
cond*elecs	(2,88):4.03/0.027	ns	ns	ns
cond*phase*elecs	ns	(2,88):4.38/0.033	ns	(2,88):4.35/0.026

The factors analyzed were: COND: comparison between inner and overt speech, PHASE: comparison between preparation and execution phase, REGION: comparison between regions, HEMI: comparison between left and right hemisphere. The numbers indicate *df, F,* and *p*-values, respectively; ns indicates non-significant.

## Supplementary Materials

The following are available online at https://www.mdpi.com/2076-3425/10/3/148/s1. Table S1: EEG results of interactions of the repeated-measure ANOVAs (first column) and post-hoc testing in the time window of 200–300 ms. Table S2: EEG results of interactions of the repeated-measure ANOVAs and post-hoc testing in the time window of 300–500 ms.
