# Supplementary material for: The Brain Differentially Prepares Inner and Overt Speech Production: Electrophysiological and Vascular Evidence
Source: Brain Sci. 2020 Mar 4;10(3):148. doi: 10.3390/brainsci10030148 (PMC7139369; doi:10.3390/brainsci10030148)
Supplement: Supplementary file 1 [file brainsci-10-00148-s001.zip › appendices-634397_revision1.docx]

**Appendices**

**A1**

| *ANOVA* | *post-hoc t-tests* | | |
| --- | --- | --- | --- |
| **cond*phase*region**  (5,22):10.05/<.0001 | F3FC3F4FC4 | (45):-3.179/.003 | preparation: inner > overt |
|  | F5FC5F6FC6 | (45):-3.069/.004 |  |
|  | C5T7C6T8 | (45):-3.530/.001 |  |
| **cond*phase*elecs**  (5,22):4.38/.033 | Fz | (44):3.37/.002 | preparation: inner > overt |
|  | Cz | (45):3.88/<.0001 |  |

**Table A1.** EEG results of interactions of the repeated-measure ANOVAs (first column) and post-hoc testing in the time window 200-300 ms. The factors analysed were: COND: comparison between inner and overt speech, PHASE: comparison between preparation and execution phase, REGION: comparison between regions. The numbers indicate *df, F/t,* and *p*-values, respectively. >: the first condition is more negative than the second one. Post-hoc testing adjusted according to Bonferroni correction. Post-hoc testing which did not survived Bonferroni are not reported in the table.

**A2**

| *ANOVA* | *post-hoc t-tests* | | |
| --- | --- | --- | --- |
| **cond*phase*region**  (5,22): 6.86/<.0001 | C3CP3C4CP4 | (45):-3.35/.002 | execution: inner > overt |
|  | CPP5HP3CPP6HP4 | (45):-4.36/<.0001 |  |
|  | P5P7P6P8 | (45):-3.73/.001 |  |
| **cond*region*hemi**  (5,22): 3.69/.012 | F4FC4 | (45):-3.41/.001 | all phase: inner > overt |
|  | C3CP3 | (45):-4.71/<.0001 |  |
|  | C4CP4 | (45):-4.07/<.0001 |  |
|  | C5T7 | (45):-3.34/.002 |  |
|  | CPP5HP3 | (45):-5.56/<.0001 |  |
|  | CPP6HP4 | (45):-3.34/.002 |  |

**Table A2.** EEG results of interactions of the repeated-measure ANOVAs and post-hoc testing in the time window 300-500 ms. The factors analysed were: COND: comparison between inner and overt speech, PHASE: comparison between preparation and execution phase, REGION: comparison between regions, HEMI: comparison between left and right hemisphere. The numbers indicate *df, F/t,* and *p*-values, respectively. >: the first condition is more negative than the second one. Post-hoc testing adjusted according to Bonferroni correction. Post-hoc testing which did not survived Bonferroni are not reported in the table.
